# Polypharmacy, potentially inappropriate medications, and drug-drug interactions in older COVID-19 inpatients

**DOI:** 10.1186/s12877-023-04487-9

**Published:** 2023-11-25

**Authors:** Zhaoyan Chen, Fangyuan Tian, Ya Zeng

**Affiliations:** 1 Department of Pharmacy, West China Hospital, Sichuan University, Chengdu, China; 2 Department of Epidemiology and Health Statistics, West China School of Public Health and West China Fourth Hospital, Sichuan University, Chengdu, China

**Keywords:** COVID-19, Potentially inappropriate medications, Polypharmacy, Older, Mortality

## Abstract

**Objectives:**

The purpose of this study was to assess the impact of polypharmacy, potentially inappropriate medications, and drug-drug interactions on in-hospital mortality in older COVID-19 inpatients.

**Methods:**

A cross-sectional study was conducted using electronic medical data from a tertiary hospital in Chengdu from December 2022 to January 2023. The 2019 AGS/Beers criteria was used to evaluate the potentially inappropriate mediation (PIM) status of older COVID-19 inpatients (age ≥ 65 years), the drug-drug interactions were evaluated on Medscape, and multivariate logistic regression was used to identify the risk factors associated with in-hospital mortality.

**Results:**

A total of 206 older COVID-19 inpatients were included in the study. The mean number of drugs per day was 13.04. The prevalence of PIM use based on the 2019 AGS Beers Criteria was 66.99%. The prevalence of drug-drug interactions was 61.65%. Logistic regression demonstrated that age ≥ 80 (OR: 10.321, 95% CI: 1.649, 64.579, *P* = 0.013), renal insufficiency (OR: 4.740, 95% CI: 1.366, 16.447, *P* = 0.014), long-term hospitalization (OR: 6.637, 95% CI: 1.030, 42.779, *P* = 0.046), severe pneumonia (OR: 50.230, 95% CI: 5.180, 487.041, *P* = 0.001) were influencing factors associated with in-hospital mortality in older COVID-19 inpatients.

**Conclusions:**

The polypharmacy, potentially inappropriate medications, and drug-drug interactions were seen in many older COVID-19 inpatients.

## Introduction

Since December 2019, the coronavirus disease 2019 (COVID-19) epidemic has been reported in various parts of the world. To date, COVID-19 continues to spread globally and poses a threat to human health [1, 2]. As the virus continues to mutate, the virulence of the mutant strain of Omicron is now weaker than at the beginning of the epidemic, but its infectiousness and immune evasion ability have increased, and the risk of becoming seriously ill after infection remains high in older patients, especially those with underlying disease [3]. The World Health Organization reported a global mortality rate of approximately 0.8% for patients aged 60 to 69 years with COVID-19, 2.2% for those aged 70 to 79 years with COVID-19, and 3.1% for those aged 80 years and older in early January 2023 [4]. More than 90% of COVID-19 related deaths in China are combined with underlying diseases, and the prognosis is worse in older patients with more than two underlying diseases combined [3].

When more than five drugs are taken at the same time, it is called polypharmacy. This multidrug combination therapy may increase the probability of drug interactions, some of which will lead to serious consequences. The part of these drugs whose potential adverse risks exceed the expected benefits is called potentially inappropriate mediation (PIM) [5]. To identify PIMs, several assessment tools have been developed, the most common being the American Geriatrics Society (AGS) Beers’ criteria. The coexistence of multiple diseases is common in older patients, and the coexistence of multiple diseases is bound to lead to the application of multiple drugs. However, the physiological function of older patients is reduced, and the changes of pharmacokinetics and pharmacodynamics lead to the increased risk of drug interaction [6], drug-drug interactions will further aggravate these adverse effects. It has been reported that polypharmacy ranks the third among the reasons for hospitalization of older patients and ranks the first among hospital-acquired diseases [7–9]. Pharmacokinetic characteristics and underlying disease use in older patients may affect the efficacy and safety of COVID-19 drugs.

At present, some studies [10, 11] have reported the polypharmacy and potentially inappropriate medications among COVID-19 inpatients in different countries. However, there is still a lack of report on drug safety in elderly COVID-19 patients in China. In addition, it is unclear about the impact of drug safety on in-hospital mortality in older COVID-19 patients. Therefore, the purpose of this study is to summarize and analyze the polypharmacy, potentially inappropriate medications, and drug-drug interactions in older COVID-19 inpatients to provide more relevant evidence for further clinical application.

## Materials and methods

### Setting and sample

A cross-sectional study was designed at the West China Hospital Sichuan University, which is a tertiary hospital of national center for the treatment of difficult and critical illnesses in west China. This research was conducted in accordance with the Helsinki World Medical Association Declaration. All data were retrospectively collected without any possibility of individual identification. The study protocol was approved by the relevant ethics review committee (2020/651).

### Data collection

The inclusion criteria were that the older patients (aged ≥ 65) with COVID-19, all of the included older patients were hospitalized for tested positive for COVID-19 virus, comprising patients admitted between December 2022 to January 2023. Finally, 206 older COVID-19 inpatients were included in our study. The exclusion criteria were that patients (aged < 65) who did not take medication during hospitalization. The primary outcome of this study was in-hospital mortality. The data were collected as follows: (1) patient characteristics (age, sex, liver function, renal function, length of hospitalization and, in-hospital mortality); (2) disease characteristics (diagnosis, comorbidities, severe pneumonia, and fungal pneumonia); and (3) medication characteristics (the number of drugs, polypharmacy, PIMs, drug-drug interactions, and anti-COVID-19 oral small molecule drugs). We identified ≥ 8 medications as the optimal cut off value for polypharmacy [12]. The 2019 Beers criteria were used to evaluate PIM use in older COVID-19 inpatients [13]. The evaluation of drug-drug interactions was through the Drug Interaction Checker of Medscape [14]. Judge by filling these medications into this website, and mild, moderate, or severe drug-drug interactions were all included. Liver and renal function were evaluated according to diagnosis. Each older patient's drugs were examined, and their polypharmacy, PIMs, drug-drug interactions were evaluated separately by two researchers. Any discrepancies between the findings of the two researchers would be brought to the attention of a third researcher, who would then propose a solution.

### Statistical analysis

The categorical variables were compared between groups using the χ2 test, and categorical data are displayed according to frequency. The mean and standard deviation (SD) are used to represent continuous data that was regularly distributed, whereas the median (M) and interquartile range (IQR) are used to express continuous data that was not normally distributed. We constructed three models: Model 1 (logistic regression with no adjustment), Model 2 (adjusted for age), and Model 3 (adjusted for age and sex). Multivariate binary logistic regression analysis included variables having statistical significance (*P* < 0.05) in univariate analysis. Using SPSS 26.0 version (IBM Corp., Armonk, NY), univariate analysis and multivariate logistic regression analysis were both performed.

## Results

### Characteristics of the study

A total of 206 older COVID-19 inpatients were included in this study, 68.93% (*n* = 142) of whom were male. The in-hospital mortality of older COVID-19 inpatients was 15.53% (*n* = 32). The oldest (≥ 80 years of age) older COVID-19 inpatients accounted for 65.05% (*n* = 134). A total of 32.52% (*n* = 67) of older inpatients had renal insufficiency, 19.42% (*n* = 40) of older inpatients had liver insufficiency. The median number of diseases was 12 (IQR: 8, 16), and 68.45% (*n* = 141) of patients had more than ten chronic diseases in addition to suffering from COVID-19. Regarding medication of patients, the median number was 12 (IQR: 8, 17), and 66.02% (*n* = 136) of patients were hyperpolypharmacy. The length of hospitalization was 16.5 (IQR: 12, 23). The proportion of oral small molecule drugs use by older COVID-19 inpatients was 45.63% (*n* = 94). 48.54% (*n* = 100) of patients had severe pneumonia, and 9.71% (*n* = 20) had fungal pneumonia. The characteristics of the basic information in this study are listed in Table 1.

**Table 1:** Basic characteristics of older COVID-19 inpatients

Characteristics	Total	Survivors group	Non-survivors group	*P* value
N (%)	206 (100.00)	174 (84.47)	32 (15.53)	
Sex, n (%)				0.101
Male	142 (68.93)	116 (66.67)	26 (81.25)	
Female	64 (31.07)	58 (33.33)	6 (18.75)	
Age, years (IQR), n (%)	84 (76, 90)			0.001
65–79	72 (34.95)	69 (39.66)	3 (9.38)	
≥ 80	134 (65.05)	105 (60.34)	29 (90.63)	
Renal insufficiency, n (%)				< 0.001
No	139 (67.48)	130 (74.71)	9 (28.13)	
Yes	67 (32.52)	44 (25.29)	23 (71.88)	
Liver insufficiency, n (%)				0.005
No	166 (80.58)	146 (83.91)	20 (62.50)	
Yes	40 (19.42)	28 (16.09)	12 (37.50)	
No. of diseases [IQR], n (%)	12 [8, 16]			0.001
3–4	5 (2.43)	5 (1.15)	0 (0.00)	
5–9	60 (29.13)	59 (33.91)	1 (3.13)	
≥ 10	141 (68.45)	110 (63.22)	31 (96.88)	
No. of medications [IQR], n (%)	12 [8, 17]			< 0.001
0–9	70 (33.98)	66 (37.93)	4 (12.50)	
10–19	100 (48.54)	88 (42.72)	12 (37.50)	
≥ 20	36 (17.48)	20 (11.49)	16 (50.00)	
Length of hospitalization [IQR], n (%)	16.5 [10, 12]			< 0.001
< 30	185 (89.81)	162 (93.10)	23 (71.88)	
≥ 30	21 (10.19)	12 (6.90)	9 (28.13)	
Type of anti-COVID-19 oral small molecule drugs, n (%)				0.151
No	112 (54.37)	90 (51.72)	22 (68.75)	
Paxlovid	76 (36.89)	69 (39.66)	7 (21.88)	
Azvudine	18 (8.74)	15 (8.62)	3 (9.38)	
Polypharmacy, n (%)				0.017
No	46 (22.33)	44 (25.29)	2 (6.25)	
Yes	160 (77.67)	130 (74.71)	30 (93.75)	
Drug-drug interactions, n (%)				0.091
No	79 (38.35)	71 (40.80)	8 (25.00)	
Yes	127 (61.65)	103 (59.20)	24 (75.00)	
PIM, n (%)				0.062
No	68 (33.01)	62 (35.63)	6 (18.75)	
Yes	138 (66.99)	112 (64.37)	26 (81.25)	
Severe pneumonia, n (%)				< 0.001
No	106 (51.46)	105 (60.34)	1 (3.13)	
Yes	100 (48.54)	69 (39.66)	31 (96.88)	
Fungal pneumonia, n (%)				< 0.001
No	186 (90.29)	164 (94.25)	22 (68.75)	
Yes	20 (9.71)	10 (5.75)	10 (31.25)	

*PIM* potentially inappropriate medication

### Prevalence of polypharmacy, potentially inappropriate medications, and drug-drug interactions

Prevalence of polypharmacy was 77.67% (*n* = 160), and the prevalence of drug-drug interactions was 61.65% (*n* = 127). Paxlovid was the most likely drug to cause drug-drug interactions (*n* = 47). Among the 206 older COVID-19 inpatient, 138 (66.99%) were identified with at least one PIM, and a total of 300 PIMs were detected by 2019 AGS Beers criteria (Table 2). Of the patient prescriptions with PIM, 36.23% were found to receive one PIM, 31.16% were found to receive 2 PIMs, and 32.61% were detected to have at least 3 PIMs according to the criteria.

**Table 2:** The number of PIMs used by older COVID-19 inpatients

Characteristics	2019 Beers Criteria
PIM inpatients	138
PIMs, n (%)	300
1PIM	50 (36.23)
2 PIMs	43 (31.16)
≥ 3 PIMs	45 (32.61)

*PIM* potentially inappropriate medication

Based on the 2019 AGS Beers Criteria, 294 medications were detected in the PIMs (Table 3). The top five medications of PIM use recognized by the criteria were diuretics (37.41%), followed by benzodiazepines and benzodiazepine receptor agonist hypnotics (24.49%), insulin (12.59%), antipsychotics (5.10%) and rivaroxaban or dabigatran (5.10%).

**Table 3:** Top 5 PIMs used by older COVID-19 inpatients in this study

Number	PIM items	Medications	*N* = 294 (%)
1	Diuretics		110 (37.41)
		Furosemide	65 (22.11)
		Spironolactone	38 (12.93)
		Hydrochlorothiazide	4 (1.36)
		Torasemide	3 (1.02)
2	Benzodiazepines, benzodiazepine receptor agonist hypnotics		72 (24.49)
		Estazolam	25 (8.50)
		Alprazolam	22 (7.48)
		Zolpidem	11 (3.74)
		Lorazepam	7 (2.38)
		Eszopiclone	6 (2.04)
		Diazepam	1 (0.34)
3	Insulin	Insulin	37 (12.59)
4	Antipsychotics		15 (5.10)
		Olanzapine	9 (3.06)
		Sertraline	2 (0.68)
		Quetiapine	2 (0.68)
		Trazodone	1 (0.34)
		Risperidone	1 (0.34)
5	Rivaroxaban or Dabigatran		15 (5.10)
		Rivaroxaban	12 (4.08)
		Dabigatran	3 (1.02)

*PIM* potentially inappropriate medication

### Risk factors for in-hospital mortality

The in-hospital mortality of polypharmacy group (23.08%) was higher than non-polypharmacy group (4.55%). The in-hospital mortality of drug-drug interactions group (23.30%) was higher than non-drug-drug interactions group (11.27%). The in-hospital mortality of PIM group (23.21%) was higher than non-PIM group (9.68%). The in-hospital mortality was the dependent variable (Survivors = 0, Non-survivors = 1), the logistic regression demonstrated that age ≥ 80 (OR: 10.321, 95% CI: 1.649, 64.579, *P* = 0.013), renal insufficiency (OR: 4.74, 95% CI: 1.366, 16.447, *P* = 0.014), long length of hospitalization (OR: 6.637, 95% CI: 1.030, 42.779, *P* = 0.046), severe pneumonia (OR: 50.23, 95% CI: 5.180, 487.041, *P* = 0.001) were positively associated with in-hospital mortality in older COVID-19 inpatients, after adjusting the foundations, the result remains the same (Table 4).

**Table 4:** Multivariate logistic regression analysis of factors associated with in-hospital mortality

Characteristics	Model 1			Model 2			Model 3		
	**OR**	**95% CI**	***P* value**	**OR**	**95% CI**	***P* value**	**OR**	**95% CI**	***P* value**
Age									
65–79	Reference								
≥ 80	10.321	1.649–64.579	0.013						
Sex, n (%)									
Male	Reference			Reference					
Female	2.114	0.493–9.069	0.314	2.462	0.597–10.150	0.212			
Renal insufficiency									
No	Reference			Reference			Reference		
Yes	4.74	1.366–16.447	0.014	4.495	1.418–14.241	0.011	4.495	1.431–14.116	0.01
Liver insufficiency									
No	Reference			Reference			Reference		
Yes	2.609	0.620–10.976	0.191	1.419	0.413–4.873	0.579	1.338	0.399–4.486	0.638
No. of diseases									
3–4	Reference			Reference			Reference		
5–9	0.999	0		0.999	0		0.999	0	
≥ 10	0.342	0.032–3.708	0.378	0.192	0.019–1.926	0.161	0.178	0.017–1.815	0.145
No. of medications									
0–9	Reference			Reference			Reference		
10–19	0.128	0.006–2.980	0.201	0.232	0.011–4.687	0.341	0.243	0.014–4.345	0.336
≥ 20	0.269	0.063–1.157	0.078	0.334	0.088–1.276	0.109	0.348	0.092–1.308	0.118
Length of hospitalization									
< 30	Reference			Reference			Reference		
≥ 30	6.637	1.030–42.779	0.046	7.927	1.283–48.989	0.026	5.791	1.107–30.3	0.038
Type of oral small molecule drugs									
No	Reference			Reference			Reference		
Paxlovid	0.503	0.043–5.947	0.585	1.077	0.121–9.588	0.947	1.123	0.137–9.233	0.914
Azvudine	0.297	0.024–3.717	0.347	0.593	0.062–5.657	0.65	0.666	0.076–5.813	0.713
Polypharmacy									
No	Reference			Reference			Reference		
Yes	1.66	0.065–42.526	0.759	1.888	0.093–38.303	0.679	1.909	0.102–35843	0.666
Drug-drug interactions									
No	Reference			Reference			Reference		
Yes	0.544	0.114–2.592	0.444	0.584	0.134–2.537	0.472	0.545	0.128–2.417	0.411
PIM									
No	Reference			Reference			Reference		
Yes	0.22	0.034–1.415	0.111	0.307	0.059–1.588	0.289	0.22	0.054–1.543	0.146
Severe pneumonia									
No	Reference			Reference			Reference		
Yes	50.23	5.180–487.041	0.001	49.445	5.266–464.309	0.001	59.485	6.312–560.586	0.001
Fungal pneumonia									
No	Reference			Reference			Reference		
Yes	3.13	0.695–14.092	0.137	2.224	0.575–8.603	0.247	2.39	0.626–9.126	0.203

Model 1: Multivariate logistic regression analysis of factors associated with in-hospital mortality in older inpatients

Model 2: Multivariate logistic regression analysis of factors associated with in-hospital mortality in older inpatients adjusted by age

Model 3: Multivariate logistic regression analysis of factors associated with in-hospital mortality in older inpatients adjusted by age and sex

*PIM* potentially inappropriate medication, *OR* odds ratio, *CI* confidence interval

## Discussion

To the best of our knowledge, this is the first innovative attempt to assess the impact of polypharmacy, PIMs, and drug-drug interactions on in-hospital mortality in older COVID-19 inpatients. The major findings were as follows: Firstly, high prevalence rates of polypharmacy (77.67%), PIMs (66.99%), and drug-drug interactions (61.65%) were detected. Secondly, diuretics, followed by benzodiazepines and benzodiazepine receptor agonist hypnotics, insulin, antipsychotics and rivaroxaban or dabigatran were the most frequently used PIMs. Thirdly, we also found that age ≥ 80, renal insufficiency, long length of hospitalization, and severe pneumonia were positively associated with in-hospital mortality in older COVID-19 inpatients.

COVID-19 vaccination has greatly changed the course of the pandemic and saved tens of millions of lives around the world. However, public immunization takes a long time because there is a problem of public acceptance of vaccines, especially for older populations [15–17]. The older population has poorer physical conditions, which was the population most affected by COVID-19 [18, 19]. The health status of COVID-19 patients was noticeably worse two years later than the overall population especially for the older [20]. As a result, it's crucial to develop an efficient treatment strategy for older COVID-19 patients [21] and to start using medications as soon as possible to treat COVID-19-related symptoms. The safety of medication administration is of concern.

Based on the 2019 AGS Beers Criteria, our study found that the prevalence of PIM use among older COVID-19 inpatients was 66.99%, which was higher than Malaysian with a prevalence of 32.7% [11] and lower than COVID-19 inpatients in Turkey reported a prevalence of 96% [10], respectively. Compared with Chinese inpatients with chronic diseases (72.54%) [22], the PIM use of older COVID-19 outpatients was lower. The universality of PIM use depends on the specific chronic diseases of patient and local prescription. Prevalence of polypharmacy was 77.67%, and the prevalence of drug-drug interactions was 61.65%. The in-hospital mortality of polypharmacy group, drug-drug interactions group, and PIM group were higher than control groups. But there was no statistical significance found in our study. This may be related to the small sample size we included in the study, the older patients, and the higher proportion of severe diseases. The older COVID-19 inpatients were typically in terrible physical and mental state, and patients' desire to accept medication was generally strong. This included not just antiviral medications but also antibiotics and sedative-hypnotic medications. Another possible explanation was that older COVID-19 patients' poor clinical outcomes, particularly in individuals with severe cases, were strongly correlated with PIM usage, which in turn increased the prevalence of PIM use. Deprescribing, which is the act of withdrawing an unsuitable medicine under the supervision of a healthcare professional, is one response to this circumstance that aims to manage polypharmacy and improve results [23]. Paxlovid is composed of two drugs, including nirmatrelvir and ritonavir, both of them are substrates of CYP3A, any drug that affects the enzyme activity of CYP3A metabolism will change the metabolism of nirmatrelvir and ritonavir, thus affecting their efficacy and safety. In addition, nirmatrelvir itself is a powerful irreversible inhibitor of CYP3A, which can increase the blood concentration of other CYP3A substrates, thereby enhancing the efficacy of combination drugs or increasing the risk of adverse reactions.

A factor analysis of older COVID-19 inpatients revealed that renal insufficiency, long length of hospitalization, and severe pneumonia, and age ≥ 80 were all strongly correlated with in-hospital mortality. Oldest COVID-19 patients often have lower health, higher multimorbidity, and are more likely to experience mortality than the overall COVID-19 group of older persons [24]. Our research showed that the risk of in-hospital mortality would steadily increase with the presence of renal insufficiency in older COVID-19 patients. To another research [25], this occurrence is comparable to Chinese individuals who have COVID-19. those with extended hospital stays had a risk of in-hospital mortality that was more than six times higher than those with short hospital stays. In addition, severe pneumonia entails a greater risk of in-hospital death in older COVID-19 inpatients, indicating that older patients were at a higher risk of mortality and longer hospitalization [26].

In addition, diuretics were the most commonly prescribed PIM in Chinese older COVID-19 inpatients, followed by benzodiazepines and benzodiazepine receptor agonist hypnotics, insulin, antipsychotics, and rivaroxaban or dabigatran. This was slightly different from our other study about Chinese inpatients with chronic diseases, in which benzodiazepines, non-steroidal anti-inflammatory drugs (NSAIDs), and antipsychotics were [22]. Insulin is commonly used in hospitalized patients. As mentioned in the 2019 AGS Beers Criteria, it is not recommended to use a sliding scale insulin formulation (rapid acting or short acting) as the only treatment for diabetes, rather than other basic insulin or long-acting insulin. Therefore, it is necessary to use this type of drug with caution.

Diuretics, which fall within the third category of the 2019 AGS Beers Criteria, were found to be the PIMs that were used the most frequently, according to our analysis. Diuretics should be administered carefully and kept at the lowest dose possible in accordance with the criteria because of their propensity to worsen or cause hyponatremia or the syndrome of inappropriate antidiuretic hormone secretion (SIADH) in elderly patients [27, 28]. In COVID-19, cachexia and low protein levels may result in systemic edema. Diuretics can therefore help to reduce edema in COVID-19. However, electrolyte levels such as sodium, potassium, and chloride should be watched during drug administration due to the adverse effects brought on by these medications, which include furosemide, hydrochlorothiazide, and spironolactone.

Our research showed that the second class of PIMs were hypnotics and benzodiazepine receptor agonists. Sleep disorders, which affect 36–70% of older persons and are typical with aging [29, 30], will worsen in older COVID-19 patients. As a result, older COVID-19 patients typically use benzodiazepines and benzodiazepine receptor agonist hypnotics. But in older adults, they are also associated with mortality, fracture, fall, and depression risks [31–33]. Therefore, for older COVID-19 patients, it is important to assess the danger of using this category of medications [34].

This study has a few issues that should be pointed out. First, the study was an observational one carried out in one center in China, there may be some restrictions on external implementation. Multi-center clinical trials must be conducted to further corroborate the conclusion. The association between polypharmacy, potentially inappropriate drugs, and drug-drug interactions and further mortality is unknown because there are no follow-up data for these older inpatients. Finally, the study's primary focus was on the inpatients of tertiary hospitals; inpatients who were in nursing homes and the community were not assessed.

## Conclusion

This study evaluated at how older COVID-19 inpatients' in-hospital mortality was affected by polypharmacy, potentially inappropriate medications, and drug-drug interactions. The results showed that polypharmacy, potentially inappropriate medications, and drug-drug interactions were seen in many older COVID-19 inpatients, and age ≥ 80, renal insufficiency, long length of hospitalization, and severe pneumonia were risk factors for in-hospital mortality.

## Data Availability

All data generated or analyzed during this study can be obtained from the corresponding author upon inquiry.
